# The interaction of vitamin D supplementation with *Omentin-1* gene polymorphism on metabolic factors and anthropometric indices in women with prediabetes: a study protocol for a double-blind randomized controlled trial

**DOI:** 10.1186/s12906-025-05034-2

**Published:** 2025-08-06

**Authors:** Roghayeh Molani-Gol, Maryam Rafraf, Mohammad Asghari Jafarabadi, Dariush Shanehbandi

**Affiliations:** 1 Student Research Committee, Tabriz University of Medical Sciences, Tabriz, Iran; 2 Nutrition Research Center, Department of Community Nutrition, Faculty of Nutrition and Food Science, Tabriz University of Medical Sciences, Attarneishabori street, Daneshghah Avenue, Tabriz, Iran; 3 Cabrini Research, Cabrini Health, Malvern, VIC 3144 Australia; 4 School of Public Health and Preventive Medicine, Monash University, Melbourne, VIC 3004 Australia; 5 Department of Psychiatry, School of Clinical Sciences, Monash University, Clayton, VIC 3168 Australia; 6 Immunology Research Center, Tabriz University of Medical Sciences, Tabriz, Iran

**Keywords:** Prediabetes, Vitamin D, Omentin-1, Polymorphism, Randomized controlled trial, Women

## Abstract

**Background:**

Prediabetes is a public health problem, and its prevalence is increasing around the world. Providing an effective strategy to prevent the progression of prediabetes and, consequently, type-2 diabetes mellitus (T2DM) could be useful for global health. Research suggests that vitamin D might contribute to decreasing the risk of developing and progressing T2DM. Moreover, *Omentin-1* Val109Asp polymorphism is also reported to be associated with insulin resistance. Therefore, the primary aim of this trial is to investigate the interaction of vitamin D supplementation with *Omentin-1* gene polymorphism on metabolic factors and anthropometric indices in women with prediabetes.

**Methods/design:**

In this double-blind randomized controlled trial, prediabetic women (*n* = 204) aged 18–65 years that will be recognized based on FBS: 100–125 mg/dL or HbA1c: 5.7-6.4% will be invited to participate in this study. After obtaining informed consent, all participants’ blood samples will be achieved to determine the Omentin-1 polymorphism (Val109Asp) genotypes. Then the women will be randomized to the intervention (*n* = 24) or placebo (*n* = 24) groups (1:1) in each genotype of Omentin-1 polymorphism. In total, 144 women will be allocated to receive vitamin D (50000 IU) or a placebo (1:1) every two weeks for 12 weeks. Supplements will be provided to the participants at the beginning of the study and the end of each month and data will be collected at the baseline and after 12 weeks. Primary outcome measures are fasting glucose, insulin serum, and serum lipid profile levels, and secondary outcome measures include anthropometric parameters and dietary intakes.

**Discussion:**

The present trial will provide more required clinical evidence on the effects of vitamin D supplementation on glycemic control and insulin resistance by considering *Omentin-1* polymorphism genetic variation in prediabetic patients, which is relevant for preventing T2DM.

**Trial registration:**

IRCT20100408003664N26.

**Supplementary Information:**

The online version contains supplementary material available at 10.1186/s12906-025-05034-2.

## Introduction

Prediabetes is a transitional state where blood sugar levels exceed the normal range but fall short of diabetes diagnosis criteria, placing individuals at elevated risk for developing diabetes and its associated complications [1]. The American Diabetes Association defines prediabetes as having a fasting blood sugar (FBS) between 100 and 125 mg/dL (impaired fasting glucose or IFG), a 2-hour glucose level between 140 and 199 mg/dL (impaired glucose tolerance), or a glycated hemoglobin (HbA1c) between 5.7 and 6.4% [1]. In 2021, the worldwide prevalence of impaired glucose tolerance (IGT) and impaired fasting glucose (IFG) was 9.1% (464 million) and 5.8% (298 million), respectively. These figures are expected to rise to 10.0% (638 million) and 6.5% (414 million) by 2045, respectively [2]. Increasing the prevalence of prediabetes as an early stage of type-2 diabetes mellitus (T2DM) is a relatively major public health concern with considerable costs, morbidity, and mortality [3, 4]. Thus, identifying preventive factors for prediabetes is crucial.

Prediabetes and obesity are closely related, since, adipose tissue secretes adipocytokines which have a vital role in the pathogenesis of insulin resistance and diabetes [5–7]. Omentin is one of the visceral adipose tissue secretory adipokines [8] which has an important role in increasing insulin sensitivity through paracrine and endocrine factors [9]. There are two highly homologous isoforms of omentin, and omentin-1 is the main form of omentin in circulation [10]. The gene encodes the omentin-1 is located in the first chromosome and involves a polymorphism at the fourth exon, which results in the replacement of asparagine with valine at position 109 of the *Omentin-1* gene (Val109Asp, rs2274907) [11]. Recent studies have investigated the association of the Val109Asp polymorphism of the *Omentin-1* gene with T2DM [12, 13]. For example, Khushi et al. in a survey of adults newly diagnosed with diabetes have shown that *Omentin-1* Val109Asp polymorphism is significantly associated with higher insulin resistance [12]. Moreover, Splichal et al. showed that the interaction of the *Omentin-1* gene Val109Asp polymorphism with BMI can reduce insulin sensitivity [14]. They also showed an association of this polymorphism with daily energy, fat, and protein intakes [14]. So, the *Omentin-1* gene polymorphism genotype and its products may be connected with prediabetes progression.

Interventions that could decrease insulin resistance, such as pharmacological therapies, weight loss, and lifestyle improvement, prevent and treat T2DM [15]. Vitamin D supplementation has been suggested as a potential method to lower the risk of diabetes [16, 17], however, the results of some interventional studies were inconsistent [18, 19]. On the other hand, recent research examining the connection between body fat percentage and vitamin D levels in the blood has revealed a strong negative correlation between obesity and serum vitamin D concentrations. These studies propose that vitamin D supplementation might indirectly influence diabetes incidence by reducing body fat mass [20, 21]. The findings of a meta-analysis demonstrated that globally, 15.7%, 47.9%, and 76·6% of participants had serum 25(OH) D levels less than 30, 50, and 75 nmol/l, respectively. Over 70% of the Iranian population also has vitamin D deficiency [22, 23]. However, a few studies have evaluated the effects of vitamin D supplement intake on prediabetic patients. To our best knowledge, no published randomized controlled trial (RCT) has investigated the effects of vitamin D supplementation in women with prediabetes by considering the Val109Asp *Omentin-1* gene polymorphism. Considering the role of omentin-1 adipokine in insulin resistance [11], investigating the effects of vitamin D in interaction with its gene polymorphism can help to provide appropriate approaches to controlling prediabetes and its health complications. Therefore, the present randomized controlled trial will be conducted to determine the interaction between vitamin D supplementation and *Omentin-1* gene polymorphism on metabolic factors and obesity indicators in women with prediabetes.

## Methods

### Study design and setting

**Fig. 1:**
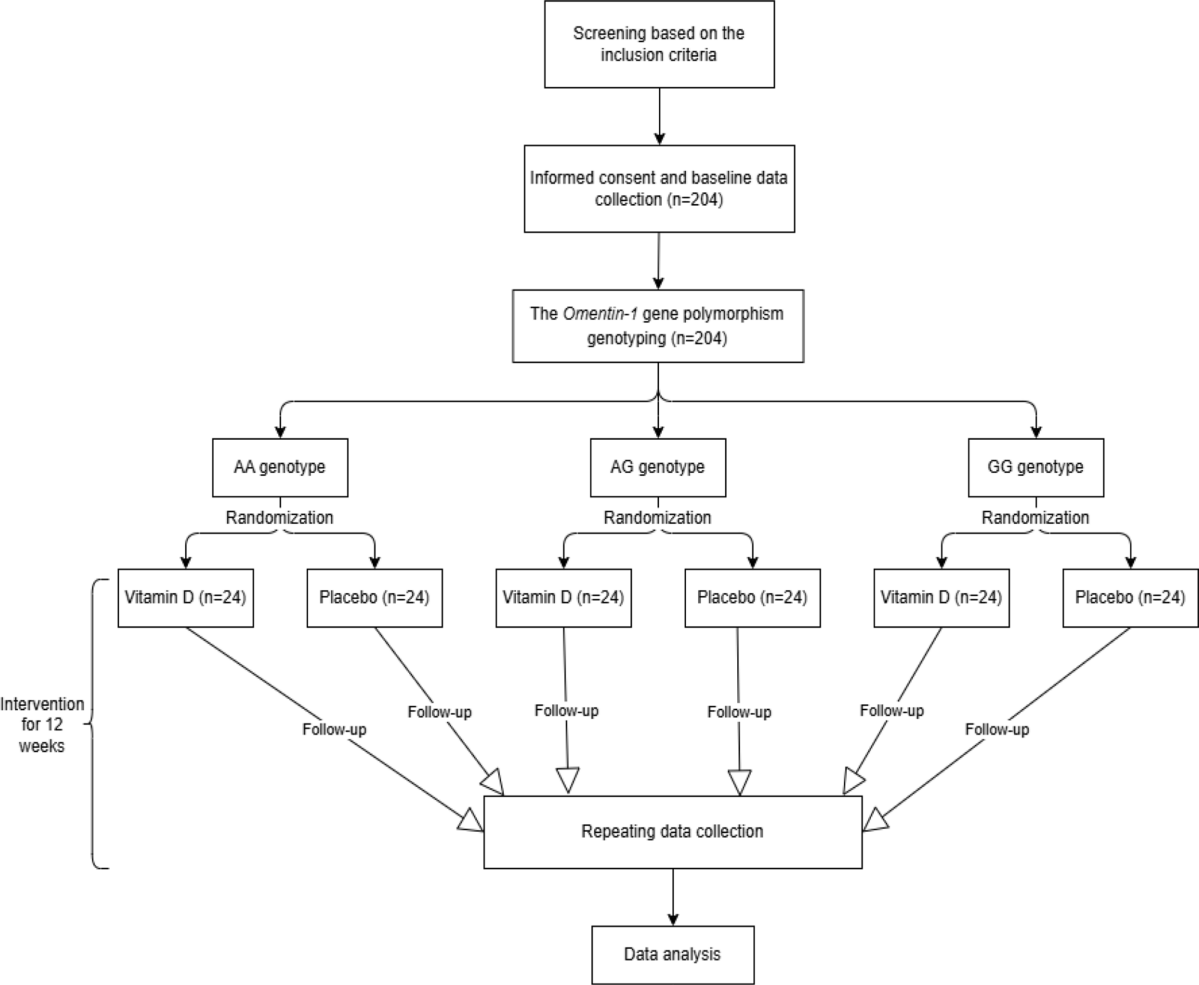
The study flow diagram

This study is a randomized, double-blind placebo-controlled trial that will be conducted on prediabetic women in Tabriz, Iran during July-December 2024. The reported protocol follows the SPIRIT checklist recommendations (Supplementary SPIRIT checklist file). The study will be carried out in two steps, the first stage will be to detect the genotypes of *Omentin-1* gene polymorphism by HRP-PCR method. In the second phase, the participants in each genotype will be randomly assigned to two groups intervention or placebo with a 1:1 allocation ratio, receiving either treatment (intervention group) or placebo (control group) for 12 weeks. The study design is presented in Fig. 1. The participants will be recruited from the healthcare center using the convenience sampling method and according to the eligibility criteria.

## Eligibility criteria

### Inclusion criteria

Women aged 18–65 years diagnosed with prediabetes who have signed an informed consent to participate. Prediabetes will be recognized based on the American Diabetes Association criteria (FBS: 100–125 mg/dL or HbA1c: 5.7-6.4%) [1].

### Exclusion criteria

Serum vitamin D levels > 100 µg/L; morbid obesity (body mass index (BMI) ≥ 40 kg/m2) or wasting (BMI < 18.5 kg/m2); pregnancy or lactation; smoking or alcohol addiction; renal failure (in dialysis); cardiovascular, thyroid, liver, or kidney diseases; cancer; chronic neurological diseases; Cushing’s syndrome; rheumatoid arthritis; surgery in the last three months; use of vitamin/nutritional supplements current or last three months; following weight loss diets; use of various drugs such as glycemic controller or lipid-lowering drugs; concomitant participation in another experimental study; and other conditions in which the researcher believes the subject is not suitable for inclusion in this study.

### Sample size

The sample size for the first and second phases will be calculated using Stata 18 software (StataCorp, College Station, Texas 77845 USA). Based on the allele frequency of the rs2274907 G > A SNP in an Iranian population [12], considering the power of 80%, alpha 0.05, and about 15% loss to follow-up, a sample of 204 participants is calculated for the first phase of the study. The sample size for the second phase, and the information obtained from Hosseini et al.‘s study [24] (FBS for the intervention group = 108.8 ± 11.5 mg/dl and for the placebo group = 99.7 ± 9 mg/dl) and with α = 0.05, and power = 0.80, is calculated to be 22 in each group, which will increase to 24 people in each group by considering about 15% loss to follow-up. Therefore, 48 women will be included in each genotype group, 24 in the intervention and 24 in the placebo groups. Therefore, 144 women will be involved in the intervention phase if sufficient participants are in each genotype.

### Ethical aspects

All procedures will be conducted following the Declaration of Helsinki and also by considering the Good Clinical Practice (GCP) guidelines [25]. In adherence to GCP principles, researchers will maintain original documents of subjects, and personal information about the participants will also be strictly confidential before, during, and after the trial to the public. Moreover, all participants will be obtained written informed consent (Supplementary files). The protocol of the current project was approved by the Ethics Committee of Tabriz University of Medical Sciences (Ethical code: IR.TBZMED.REC.1402.618) and is registered in the Iranian Registry of Clinical Trials (Registration number: IRCT20100408003664N26). Finally, the findings from this research will be submitted to international, peer-reviewed journals for publication.

At the end of the trial, if someone requires post-trial care or suffers harm from trial participation, healthcare will be provided freely for them, and financial aspects will be compensated. Moreover, the trial results will be communicated to the participants and healthcare professionals.

### Randomization, concealment, and blinding

The allocation ratio is decided to 1:1 and the participants will be randomly divided into the intervention or placebo groups, according to a computer-generated randomization plan. Stata 18 (StataCorp, College Station, Texas 77845 USA) with the ralloc methods will be used for generating the randomization sequence by random permuted block procedure with a size of 2. Then, organized in sealed opaque envelopes by a person who is not one of the investigators. The assignment sequence will be concealed from the researcher before the randomization. Participants and researchers will be blinded to the groups’ allocation.

### Intervention

**Fig. 2:**
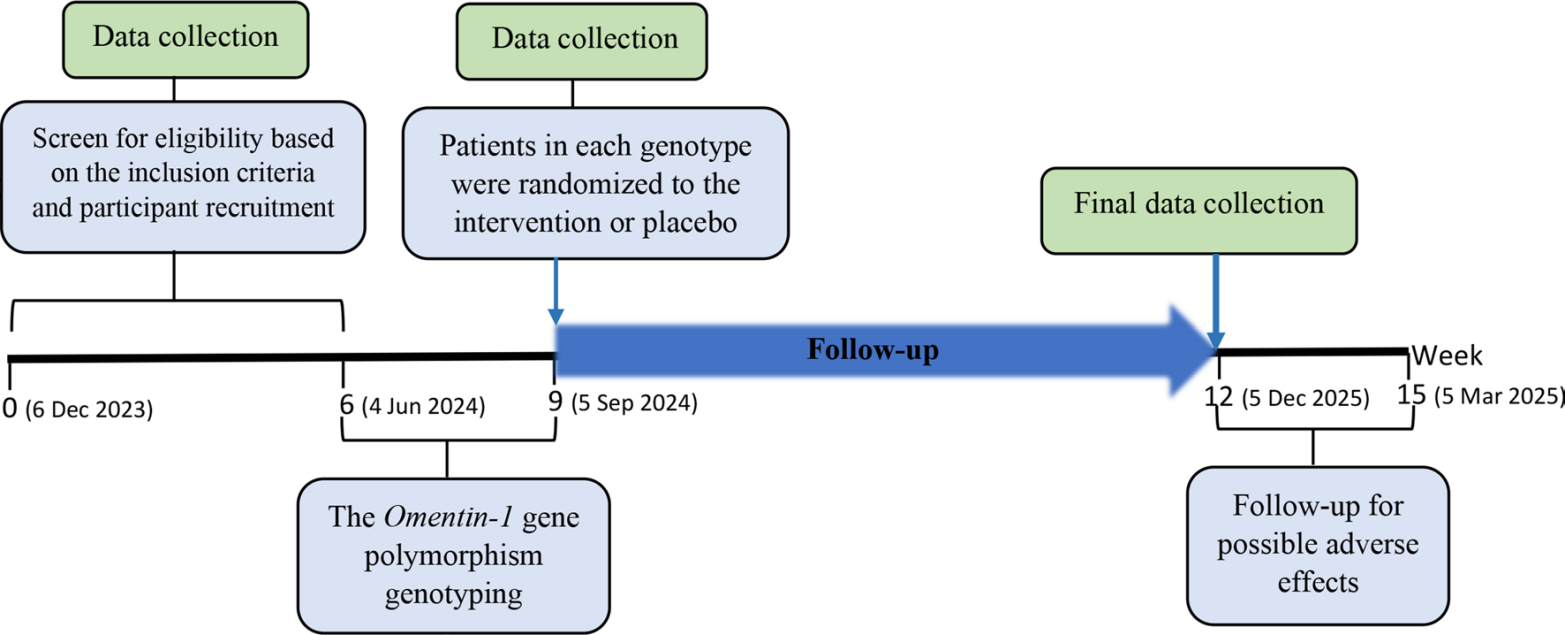
The study timeline

Individuals in the intervention groups will take a vitamin D pearl with a dose of 50,000 units every two weeks for 12 weeks. Placebo is sunflower oil that similar to the intervention will take one pearl every two weeks. The shape, size, and color of vitamin D pearls and placebo will be the same. Vitamin D and placebo pearls will be provided by Zahravi Pharmaceutical Company (Zahravi Pharmaceutical Co, Tabriz, Iran, web address: https://www.zahravi.com/en/). Supplements will be delivered to the participants at the beginning of the study and the end of each four weeks and the follow-up visits will also be carried out every four weeks (Fig. 2). The participants’ compliance with the study procedure will be tracked by recursive pearls and serum vitamin D levels change after the intervention period. Moreover, the participants’ supplement consumption will be followed up every two weeks by telephone call.

## Outcome measures

### Primary outcome

The primary outcome measures will consist of the frequency of *Omentin-1* gene polymorphism genotypes and the changes in serum levels of glycemic indices (FBS, serum insulin, and Homeostatic Model Assessment of Insulin Resistance (HOMA-IR)), lipid profiles (LDL-C, HDL-C, TG, and TC), omentin, and 25(OH)D from the baseline to 12 weeks.

### Secondary outcomes

Secondary outcome measures will be the changes in anthropometric indexes including weight, BMI, waist and hip circumferences, waist-to-height ratio (WHtR), waist-to-hip circumference (WHR), and body composition alteration from baseline to 12 weeks.

### Data collection

A structured and content and face validated (by the research team) questionnaire checklist will be administered to collect information regarding the general characteristics, and medical and treatment history (Supplementary files). Moreover, the duration of sunlight exposure during a day will be obtained by a questionnaire designed for this purpose (Supplementary files). The participants will be asked to answer questions about their average sun exposure hours on a usual day, duration of exposure, time of day when exposure occurred, how often sunscreen or sun hats were used, and to describe the clothing degree worn when outside.

The assessment of physical activity levels will be conducted using a short version of the International Physical Activity Questionnaire (IPAQ) [26] which has been translated and validated for use in Persian (Supplementary files). This survey requires participants to indicate the number of days, hours, and minutes they engaged in strenuous activities, such as aerobics, or moderate activities, like carrying lightweight objects, during the previous week. Additionally, it asks about the number of days, hours, and minutes participants walked for a minimum of 10 min, as well as the duration of time they spent seated for any activity over the last 7 days.

The women’s dietary intake will be recorded using a 24-hour dietary recall for three days (two regular days and one weekend) at the beginning and end of the study (Supplementary files). Then, these three-day recalls will be analyzed by Nutritionist-4 software, and the three-day mean caloric and nutrient intakes of participants will be estimated.

Anthropometric measurements will be recorded at the start and the end of the supplementation period. A digital platform scale will be used to measure weight, with participants standing barefoot in minimal clothing at the center. The same scale will be utilized for height measurement, with subjects standing barefoot, arms hanging freely at their sides, and palms facing their thighs. Body Mass Index (BMI) will be determined by dividing weight in kilograms by height in meters squared. To measure waist circumference (WC), a flexible plastic tape will be placed at the midpoint between the lower costal margin and iliac crest, perpendicular to the body. During this measurement, the participant will stand with feet approximately 20 cm apart and arms hanging freely. Hip circumference will be assessed at the widest point over the buttocks, with the researcher kneeling beside the participant to accurately observe the maximum extension level. WHR will be calculated by dividing waist circumstance (cm) by hip circumstance (cm) and WHtR by dividing WC by height (m). Additionally, the body composition (fat-free mass (FFM) and fat mass (FM)) of the participants will be measured by the bioelectrical impedance analysis method using Tanita’s body composition analyzer.

Blood samples will be collected after 8–12 h of fasting by a professional individual at the baseline and after 12 weeks. About 2 cc of the obtained blood sample will be used to determine the Val109Asp polymorphism of the *Omentin-1* gene and the remaining will be centrifuged at 22 °C, 5000 rpm, for 6 min and the serum will be stored in microtubes at − 70 °C for later analysis.

The Omentin-1 Val109Asp polymorphism genotypes will be recognized by the high-resolution melting-polymerase chain reaction (HRM-PCR) method. HRM technique is a new method to identify mutations (polymorphisms), in which various genotypes are identified through the difference in the melting curve. First, the genomic DNA of participants will be extracted from the whole blood using a standard phenol/chloroform method. Second, the PCR amplification of the rs2274907 will be conducted using the forward: CCCTCACCCGAGTGGGTA and reverse: GAATGACATGCGTGGGAAG primers. Then, the melting curve will be produced using the real-time device and the fluorescent color Eva green, which is more sensitive than other colors such as Cyber green. The following items will be used for the HRM procedure: 4 µl of EvvaGreen PCR Master mix 5x, 0. 5 µl (each) of Primer (4pmol/µl), 50 ng of DNA, and Up to 20 µl of H2O.

Glycemic indices including FBS, serum insulin, and HOMA-IR, which FBS will be evaluated by the enzymatic colorimetric method (kit: Pars Azmoon, Tehran, Iran), and serum insulin will be evaluated by enzyme-linked immunosorbent assay (ELISA) technique (Monobind Inc, Lake Forest, CA, USA). The HOMA-IR will be calculated using the formula (HOMA-IR= [Fasting Insulin (µg/ml) * Fasting Glucose (mmol/l)]/22.5).

Lipid profiles including total cholesterol (TC), high-density lipoprotein cholesterol (HDL-C), and serum triglycerides (TG) will be assessed by the enzymatic colorimetric method (kit: Pars Azmoon, Tehran, Iran). Low-density lipoprotein cholesterol (LDL-C) will be calculated applying the Friedewald formula: LDL-C = TC (mg/dl) _ ([HDL-C (mg/dl) + TG (mg/dl)]/5). Serum omentin-1 and 25(OH)D levels will be evaluated using the ELIZA method (Monobind kit, Monobind Inc., Lake Forest, CA, USA).

### Statistical analyses

At the end of data collection, data will be assessed using IBM SPSS Statistics software (version 20, IBM Corp, Armonk, USA). Normally distributed numerical variables will be expressed as mean ± standard deviation and non-normally distributed continuous variables will be expressed as median and interquartile range. Categorical variables will be expressed as absolute and relative frequencies. Using the t-test or Wilcoxon rank-sum test quantitative indicators will be compared among the intervention and placebo groups. Qualitative variables will also be assessed using Fisher’s exact test. All statistical tests will be significant if *p* < 0.05 (two-tailed).

To determine the effect of vitamin D supplementation and placebo on outcomes indicators among different genotypes of the *Omentin-1* gene polymorphism will be used: (1) mixed model ANCOVA or non-parametric ANCOVA (quantile regression) taking into account baseline value and confounding factors (2) mixed model ANCOVA subgroup analysis among polymorphisms with considering the interaction between the interventions and polymorphisms, and calculating the mean difference and percentage of change as the effect size. (3) two-way mixed model ANOVA with repeated measures analysis will be used to consider the effect of the intervention, measurements, and their interactions.

### Criteria for discontinuing

All women will be asked to inform the researchers if there are any changes in their usual diet, physical activity, and lifestyle. The participants will be assessed for any possible complications at each follow-up visit and the treatment will be discontinued if the following criteria are met:

1. The participant is unwilling to continue the trial and withdraws the informed consent.
2. The participant reports a serious adverse event.
3. The condition of the participant is considerably deteriorated, and she is not appropriate to continue the trial based on the investigators’ judgment.
4. The participant seriously violates the trial protocol or has poor compliance.

## Discussion

Currently, chronic diseases have become a public health problem around the world due to lifestyle changes [7]. Glucose metabolism disorder, which is a chronic metabolic condition, is known as one of the most important risk factors for the development and complications of various metabolic diseases [27]. The prevalence of prediabetes and consequently T2DM are increasing worldwide and are projected to rise to 10.0% in 2045 [2]. The results of a recent study in Iran also showed that the prevalence of prediabetes was 18.22% [3] which is more than its global prevalence. It has been shown that 70% of prediabetes patients progress to T2DM if not treated, and the annual conversion rate of prediabetes to T2DM is estimated between 5 and 10% [4].

The present RCT will examine the effects of the vitamin D intervention on glycemic indices, lipid profiles, and weight management in prediabetic women by considering the genotypes of *Omentin-1* gene polymorphism. This will be one of the first rigorously evaluated interventions that aim to address the role of genetic variation on vitamin D influences in the prevention of T2DM in prediabetic women. It has shown that Val109Asp polymorphism genetic variation may alter insulin metabolism and insulin resistance and play a key role in the development of T2DM [12, 28]. It is well known that genetic factors as same as lifestyle contribute to the development and progression of prediabetes [29, 30]. In other words, genetic predisposition is a well-studied risk factor for obesity [31], which in turn, is a major risk factor for T2DM [32]. The *Omentin* gene has recently been identified, and its product is secreted by visceral adipose tissue. The literature demonstrated that Val109Asp polymorphism could be accompanied by T2DM and obesity [10, 28, 33]. The relationship between *Omentin-1* Val109Asp genetic variations and newly diagnosed diabetes is not well determined. Previously, Khoshi et al. investigated this polymorphism in some patients with T2DM from an Iranian population and revealed a significant relationship between the *Omentin-1* gene Val109Asp polymorphism and T2DM in the studied population [12]. Moreover, they found that polymorphism of the *Omentin-1* gene (Val109Asp) was significantly related to insulin resistance, overweight/obesity, and familial history of diabetes [12]. Furthermore, other studies demonstrated that omentin levels and the Val109Asp polymorphism are associated with BMI and fatty liver disease [33, 34]. It is hypothesized that the *Omentin-1* gene Val109Asp polymorphism may contribute to lipid accumulation in the liver. Others also reported that serum omentin-1 as a visceral fat depot-specific protein has an inverse correlation with insulin resistance and obesity [9, 35]. Some studies have shown that omentin-1 via increasing insulin sensitivity enhances glucose uptake in human adipocytes [9, 11].

More than 80% of people with T2DM are classified as overweight or obese [36]. On the other hand, insulin resistance plays a crucial role in the development of T2DM which is common in obesity. Strategies that improve insulin sensitivity, such as weight reduction, changes in lifestyle, and medication-based approaches, are effective in both preventing and managing T2DM [15]. It is crucial to develop new and effective strategies for the primary prevention of T2DM that focus on decreasing insulin resistance. Some RCTs evaluated the effects of vitamin D supplementation on the prevention of prediabetes progress to T2DM in individuals with impaired blood glucose [16, 18, 37, 38]. Most of these researches indicated that the supplementation of vitamin D could reduce the risk of T2DM [16, 18, 38]. Previous epidemiological surveys among diabetic adults demonstrated that serum glucose levels are inversely related to serum 25-hydroxyvitamin D (25(OH) D) level and low 25(OH) D has emerged as a possible risk factor for T2DM [16, 17].

Vitamin D may influence insulin secretion and sensitivity through both direct and indirect pathways. Research has demonstrated that vitamin D enhances the transcription and expression of the insulin receptor gene, promoting the oxidation of glucose in both basal and insulin-stimulated conditions, thereby improving insulin sensitivity [39, 40]. Additionally, vitamin D’s regulation of extracellular calcium contributes to improved insulin action and signal transduction [41]. Furthermore, vitamin D affects β-cell insulin secretion by increasing intracellular calcium, which plays a role in β-cell glycolysis and glucose signaling [42]. It also has the potential to modulate cytokine-mediated β-cell apoptosis, a crucial factor in the development and progression of T2DM [43, 44]. Importantly, vitamin D counteracts the detrimental effects of advanced glycation products, which are linked to the development of insulin resistance and T2DM complications [45]. The discovery of vitamin D receptors and vitamin D binding protein in pancreatic islets and numerous inflammatory cells suggests that vitamin D plays a significant role in the function of these cells and the subsequent development of T2DM [46]. However, high-quality RCTs that support these findings remain scarce, thus this clinical trial aims to address this gap by conducting a comprehensive investigation of vitamin D’s effects by considering genetic variation.

If it is proven that there is an interaction between vitamin D treatment and *Omentin-1* gene polymorphism on metabolic parameters and anthropometric parameters and the intervention is effective in improving these factors among prediabetic patients, it will help fill an important gap in healthcare service delivery. The information obtained from this trial can be used to develop public health policies related to the prevention of T2DM in patients with impaired glucose levels.

### Trial status

The trial started on 6 December 2023 and it is anticipated to be completed by 5 March 2025.

## Electronic supplementary material

Below is the link to the electronic supplementary material.

Supplementary Material 1

Supplementary Material 2

Supplementary Material 3

## Data Availability

No datasets were generated or analysed during the current study.
