# Supplementary material for: The interaction of vitamin D supplementation with Omentin-1 gene polymorphism on metabolic factors and anthropometric indices in women with prediabetes: a study protocol for a double-blind randomized controlled trial
Source: BMC Complement Med Ther. 2025 Aug 6;25:299. doi: 10.1186/s12906-025-05034-2 (PMC12330007; doi:10.1186/s12906-025-05034-2)
Supplement: Supplementary file 1 — Supplementary Material 1 [file 12906_2025_5034_MOESM1_ESM.docx]

**Supplementary files:**

**Informed consent**

ID: ……… First and last name: ….................... Date: ……………

Research title:

Investigating the interaction of vitamin D supplementation with *Omentin-1* gene polymorphism on metabolic factors and anthropometric indices in women with prediabetes

Please tick:

1- I confirm that the participant information sheet dated …………. I have read and understood it for conducting the above research and I have been given the opportunity to ask the questions I want.

2- I know that my participation in this research is voluntary. I also understand that I can withdraw from the investigation at any time without being required to provide a reason.

3- I agree to participate in the above study/research.

Participant's name Date: Signature

Researcher's name Date: Signature

**General questionnaire**

ID: ……… Date: …........... Phone number: ……………

1. What is your first and last name?

………………………………..

2. How do you define your gender?

□ Male □ Female □ Other

2. How old are you?

……………. years

3. Are you pregnant or are you planning to become pregnant in the next 3 months?

□ Yes □ No

4. Are you a nursing mother?

□ Yes □ No

5. What is the highest degree or level of education you have completed?

□ Illiterate □ Under-high school □ High school □ Associate degree □ Bachelor □ Master's degree and above

6. What is your job?

……………………………..

7. Do you smoke?

□ Yes □ No

8. Have you used a special supplement in the last 3 months?

□ Yes (what kind of supplement: .........................) □ No

9. Are you currently following a special diet under the supervision of a nutritionist or doctor?

□ Yes □ No

10. Do you currently have any other disease besides pre-diabetes?

□ Yes □ No

If it is positive, which of the following diseases do you have?

□ The digestive system chronic diseases □ Cardiovascular diseases

□ Kidney diseases □ Liver diseases □ Hypertension

Other diseases....................................

11. Is there a history of diabetes in your first-degree relatives (father, mother, siblings)?

□ Yes □ No

12. What medicines are you currently taking and how much?

……………………….

**Sunlight exposure questionnaire**

ID: ……… First and last name: ….................... Date: ……………

1) Approximately how long are you exposed to direct sunlight during the day?

□ Less than 1 hour □ More than 1 hour

2) Duration of exposure to the sun during the day:

□ I don't stand in front of the sun □ 10 minutes to 1 hour

□ 1 to 2 hours □ More than 2 hours

3) What time of day and for how long is your exposure to sunlight?

□ In the morning, duration: ..............minutes per day

□ Around noon, duration: ...............minutes per day

□ In the evenings, duration: ................minutes per day

4) What parts of your body are usually exposed to the light when exposed to sunlight?

□ Face □ Hand from the wrist down □ Hand from the arm down □ Legs

5) Do you use sunscreen when exposed to sunlight? □ Yes SPF………

□ No □ Sometimes □ Often □ Always

6) When exposed to the sun, how often do you use a sun hat for protection?

□ Always □ Often □ Sometimes □ Rarely □ Never

**International physical activity questionnaire (short version)**

ID: ……… First and last name: ….................... Date: ……………

Think about all the vigorous activities that you did in the last 7 days. Vigorous physical activities refer to activities that take hard physical effort and make you breathe much harder than normal. Think only about those physical activities that you did for at least 10 minutes at a time.

1. During the last 7 days, on how many days did you do vigorous physical activities like heavy lifting, digging, aerobics, or fast bicycling?

_____ days per week

□ No vigorous physical activities

2. How much time did you usually spend doing vigorous physical activities on one of those days?

_____ hours per day

_____ minutes per day

□ Don’t know/Not sure

Think about all the moderate activities that you did in the last 7 days. Moderate activities refer to activities that take moderate physical effort and make you breathe somewhat harder than normal. Think only about those physical activities that you did for at least 10 minutes at a time.

3. During the last 7 days, on how many days did you do moderate physical activities like carrying light loads, bicycling at a regular pace, or doubles tennis? Do not include walking.

_____ days per week

□ No moderate physical activities

4. How much time did you usually spend doing moderate physical activities on one of those days?

_____ hours per day

_____ minutes per day

□ Don’t know/Not sure

Think about the time you spent walking in the last 7 days. This includes at work and at home, walking to travel from place to place, and any other walking that you have done solely for recreation, sport, exercise, or leisure.

5. During the last 7 days, on how many days did you walk for at least 10 minutes at a time?

_____ days per week

□ No walking

6. How much time did you usually spend walking on one of those days?

_____ hours per day

_____ minutes per day

□ Don’t know/Not sure

The last question is about the time you spent sitting on weekdays during the last 7 days. Include time spent at work, at home, while doing course work and during leisure time. This may include time spent sitting at a desk, visiting friends, reading, or sitting or lying down to watch television.

7. During the last 7 days, how much time did you spend sitting on a week day?

_____ hours per day

_____ minutes per day

□ Don’t know/Not sure

**Dietary 24-hour recall questionnaire**

ID: ……… First and last name: ….................... Date: ……………

| Meal | Name of consumed food | Components of consumed food | Amount of consumed food |
| --- | --- | --- | --- |
| Breakfast |  |  |  |
| Snack |  |  |  |
| Lunch |  |  |  |
| Snack |  |  |  |
| Dinner |  |  |  |
| Late night meal |  |  |  |
