# Supplementary material for: The interaction of vitamin D supplementation with Omentin-1 gene polymorphism on metabolic factors and anthropometric indices in women with prediabetes: a study protocol for a double-blind randomized controlled trial
Source: BMC Complement Med Ther. 2025 Aug 6;25:299. doi: 10.1186/s12906-025-05034-2 (PMC12330007; doi:10.1186/s12906-025-05034-2)
Supplement: Supplementary file 3 — Supplementary Material 3 [file 12906_2025_5034_MOESM3_ESM.pdf]

Protocol summary

General information

Secondary Ids

Ethics committees

Health conditions studied

Primary outcomes

Secondary outcomes

Intervention groups

Recruitment centers

Sponsors / Funding sources

Person responsible for general inquiries

Person responsible for scientific inquiries

Person responsible for updating data

Sharing plan

The effects of vitamin D supplementation on metabolic factors and obesity indices among omentin gene polymorphism genotypes in women with prediabetes

More options

Protocol summary

Study aim

The effects of vitamin D supplementation on metabolic factors and obesity indices among omentin gene polymorphism genotypes in women with prediabetes

Design

A randomized, parallel-group, double-blind, placebo-controlled trial on 48 prediabetic patients in each genotype group. The software-generated random was used for randomisation.

Settings and conduct

The patients will be selected from the endocrinology clinic of Imam Reza Hospital in Tabriz and the healthcare centers of Tabriz. Individuals were randomly allocated into vitamin D or placebo groups. The duration of the trial will be 12 weeks. The blinding will be double-blind and the participants and researcher will remain unaware until the end of the study.

Participants/Inclusion and exclusion criteria

A total of 48 patients with prediabetes will be selected based on the inclusion criteria and informed written consent will be obtained from all participants before enrollment. Inclusion criteria: willingness to participate in the study, women aged 18 to 65 years with prediabetes and BMI: 18-40 Kg/m2; Exclusion criteria:

	Serum vitamin D level higher than 100 micrograms per liter; Smoking and alcohol; Pregnancy and lactation; Diabetes and infectious heart disease; Renal failure; Cushing's syndrome; Liver disease; Use of drugs that affect the level of fasting blood sugar
**Intervention groups**	1. Vitamin D supplement group (50000 units once every two weeks) 2. Placebo group
**Main outcome variables**	glycemic indices; serum level of lipid profile; serum omentin; anthropometric measurements; vitamin D serum

General information

**Reason for update**	
**Acronym**	
**IRCT registration information**	IRCT registration number: **IRCT20100408003664N26**<br>Registration date: **2023-12-19, 1402/09/28**<br>Registration timing: **registered_while_recruiting**<br><br>Last update: **2023-12-19, 1402/09/28**<br>Update count: **0**
**Registration date**	2023-12-19, 1402/09/28
**Registrant information**	**Name** Maryam Rafraf<br>**Name of organization / entity** Tabriz University Of Medical Sciences<br>**Country** Iran (Islamic Republic of)<br>**Phone** +98 41 1335 7580

Email address rafrafm@tbzmed.ac.ir

**Recruitment status**	**Recruitment complete**
**Funding source**	
**Expected recruitment start date**	2023-12-06, 1402/09/15
**Expected recruitment end date**	2024-03-19, 1402/12/29
**Actual recruitment start date**	*empty*
**Actual recruitment end date**	*empty*
**Trial completion date**	*empty*
**Scientific title**	The effects of vitamin D supplementation on metabolic factors and obesity indices among omentin gene polymorphism genotypes in women with prediabetes
**Public title**	The effects of vitamin D supplementation on metabolic factors and obesity indices among omentin gene polymorphism genotypes in women with prediabetes
**Purpose**	Treatment
**Inclusion/Exclusion criteria**	**Inclusion criteria:**<br>Willingness to cooperate Women aged 18 to 65 years with prediabetes Body mass index in the range of 30-40 Kg/m2<br>**Exclusion criteria:**<br>Serum Vitamin D Levels higher than 100 μg / L Smoking and alcohol Pregnancy and lactation Intake of various drugs, including drugs affecting blood sugar

	and lipid levels Intake of drugs or dietary supplements affecting the body weight Surgery in last three months Having chronic neurological diseases, diabetes, cardiovascular, liver, kidney, Cushing's syndrome, rheumatoid arthritis, cancer and other diseases
**Age**	From **18 years** old to **65 years** old
**Gender**	Female
**Phase**	3
**Groups that have been masked**	• Participant<br>• Investigator
**Sample size**	Target sample size: **48**
**Randomization (investigator's opinion)**	Randomized
**Randomization description**	The eligible participants will be randomly allocated to intervention and placebo groups using software-generated random permuted blocks size of 4. The generated random sequence will be kept in a protected location and administered by an independent third party blind to the trial throughout the study. To hide, a random sequence will not be provided to the executor and will be performed sequentially for each block.
**Blinding (investigator's opinion)**	Double blinded
**Blinding description**	The placebo and supplement will be packed in the same number in similar packages. The method of blindness will be that the supplements and placebo will be delivered to the participants by someone other than the researcher, and the researcher will remain unaware until the end of the study.

**Placebo**	Used
**Assignment**	Parallel
**Other design features**	

Secondary Ids

empty

Ethics committees

1

**Ethics committee**	**Name of ethics committee** Ethics Committee of Tabriz University of Medical Science<br>**Street address** Tabriz University of Medical Science, Attar Neishabouri Avenue, Golgasht street, Tabriz<br>**City** Tabriz<br>**Province** East Azarbaijan<br>**Postal code** 5138663134
**Approval date**	2023-11-23, 1402/09/02
**Ethics committee reference number**	IR.TBZMED.REC.1402.618

Health conditions studied

1

**Description of health condition studied**	Prediabetes
**ICD-10 code**	R73.0
**ICD-10 code description**	Abnormal glucose tolerance test

Primary outcomes

1

**Description**	Glycemic indices
**Timepoint**	Baseline and 12 weeks after the intervention
**Method of measurement**	Glycemic indexes (from indicators of metabolic status) including fasting blood sugar by enzymatic method, insulin by ELISA and insulin resistance by formula, will be measured or calculated.

2

**Description**	lipid profile
**Timepoint**	Baseline and 12 weeks after the intervention
**Method of measurement**	Lipid profile (from indicators of metabolic status) including total cholesterol, triglycerides and high-density lipoprotein will be measured enzymatically, and low-density lipoprotein will be calculated using the friedwald formula.

3

**Description**	Omentin

**Timepoint**	Baseline and 12 weeks after the intervention
**Method of measurement**	ELISA

4

**Description**	Serum Vitamin D
**Timepoint**	Baseline and 12 weeks after the intervention
**Method of measurement**	ELISA

Secondary outcomes

1

**Description**	Obesity indices
**Timepoint**	Baseline and 12 weeks after the intervention
**Method of measurement**	Obesity indices including weight with scales and waist circumference and hip circumference will be measured with a tape measure. Body mass index will be calculated with the formula.

2

**Description**	Body composition
**Timepoint**	Baseline and 12 weeks after the intervention
**Method of measurement**	Body composition will be measured by Bio impedance (BIA) method usingTanita body composition analyzer.

3

**Description**	Dietary intake
**Timepoint**	Baseline and 12 weeks after the intervention

**Method of measurement**	The food record questionnaire will be used to assess food intake including energy, carbohydrate, protein and fat.

Intervention groups

1

**Description**	Intervention group: 50000 units once every two weeks vitamin D of Zahravi company for 3 month
**Category**	Treatment - Drugs

2

**Description**	Control group: Intake a Placebo perl containing sunflower oil, which is similar to vitamin D supplement in terms of dosage, color and size, once every two weeks for 12 weeks
**Category**	Placebo

Recruitment centers

1

Recruitment center

**Name of recruitment center**	Endocrinology clinic of Imam Reza Hospital , Tabriz
**Full name of responsible person**	Roghayeh Molani-Gol

**Street address**	Imam Reza Hospital, Golghast Street, Tabriz
**City**	Tabriz
**Province**	East Azarbaijan
**Postal code**	6717839391
**Phone**	+98 914 450 1684
**Email**	molanigol@tbzmed.ac.ir

Sponsors / Funding sources

1

Sponsor

**Name of organization / entity**	Tabriz University of Medical Sciences
**Full name of responsible person**	Dr.Alireza Ostadrahimi
**Street address**	Faculty of Nutrition and Food Sciences, Tabriz University of Medical Sciences, Attar Neyshabori avenue, Golgasht street, Tabriz
**City**	Tabriz
**Province**	East Azarbaijan
**Postal code**	5166614711

Phone +98 41 3336 3430
Email nut-rc@tbzmed.ac.ir

**Grant name**	
**Grant code / Reference number**	
**Is the source of funding the same sponsor organization/entity?**	Yes
**Title of funding source**	Tabriz University of Medical Sciences
**Proportion provided by this source**	100
**Public or private sector**	Public
**Domestic or foreign origin**	Domestic
**Category of foreign source of funding**	*empty*
**Country of origin**	
**Type of organization providing the funding**	Academic

Person responsible for general inquiries

Contact

Name of organization / entity Tabriz University of Medical Sciences
Full name Roghayeh Molani-Gol

**of responsible person**	
**Position**	PhD Student of Nutrition Sciences
**Latest degree**	Master
**Other areas of specialty/work**	Nutrition
**Street address**	Faculty of Nutrition and Food Sciences, Tabriz University of Medical Sciences, Attar Neyshabori Avenue , Golgasht Street, Tabriz
**City**	Tabriz
**Province**	East Azarbaijan
**Postal code**	5178741516
**Phone**	+98 41 3440 0334
**Email**	molanigol@tbzmed.ac.ir

Person responsible for scientific inquiries

Contact

**Name of organization / entity**	Tabriz University of Medical Sciences
**Full name**	Maryam Rafraf

of responsible person	
Position	Ph.D in Nutrition Sciences
Latest degree	Ph.D.
Other areas of specialty/work	Nutrition
Street address	Faculty of Nutrition and Food Sciences, Tabriz University of Medical Sciences, Attar Neyshabori Avenue , Golgasht Street, Tabriz
City	Tabriz
Province	East Azarbaijan
Postal code	5166614711
Phone	+98 41 3440 0334
Email	rafrafm@tbzmed.ac.ir

Person responsible for updating data

Contact

Name of organization / entity	Tabriz University of Medical Sciences
Full name of	Roghayeh Molani-Gol

**responsible person**	
**Position**	PhD Student of Nutrition
**Latest degree**	Master
**Other areas of specialty/work**	Nutrition
**Street address**	Faculty of Nutrition and Food Sciences, Tabriz University of Medical Sciences, Attar Neyshabori Street, Golgasht Ave, Tabriz
**City**	Tabriz
**Province**	East Azarbaijan
**Postal code**	5178741516
**Phone**	+98 41 3440 0334
**Email**	molanigol@tbzmed.ac.ir

Sharing plan

**Deidentified Individual Participant Data Set (IPD)**	Undecided - It is not yet known if there will be a plan to make this available
**Study Protocol**	Undecided - It is not yet known if there will be a plan to make this available
**Statistical Analysis Plan**	Undecided - It is not yet known if there will be a plan to make this available
**Informed Consent Form**	

	Undecided - It is not yet known if there will be a plan to make this available
**Clinical Study Report**	Undecided - It is not yet known if there will be a plan to make this available
**Analytic Code**	Undecided - It is not yet known if there will be a plan to make this available
**Data Dictionary**	Undecided - It is not yet known if there will be a plan to make this available

Tel:
Working hours:
8:00 - 15:30 Tehran time
11:30 - 19:00 GMT

0098 21 8670 5503

During COVID-19 Epidemic at working times:
0098 936 770 7834

Fax:
0098 21 8670 5503

Email:
irct@behdasht.gov.ir (mailto:admin@irct@behdasht.gov.ir)

Directly contacting the manager:
0098 912 778 2686

Address:
IRCT administration team,
Central Library Building, Iran University Campus,
Hemmat freeway, next to Milad tower,
Tehran, 14496-14535
Iran

Copyright © IRCT 2008-2025. All Rights Reserved.
